# A Simple Scatter Reduction Method in Cone-Beam Computed Tomography for Dental and Maxillofacial Applications Based on Monte Carlo Simulation

**DOI:** 10.1155/2018/5748281

**Published:** 2018-01-03

**Authors:** Chalinee Thanasupsombat, Saowapak S. Thongvigitmanee, Sorapong Aootaphao, Pairash Thajchayapong

**Affiliations:** ^1^X-Ray CT and Medical Imaging Laboratory, National Electronics and Computer Technology Center, National Science and Technology Development Agency, 112 Thailand Science Park, Paholyothin Road, Khlong Nueng, Khlong Luang, Pathum Thani 12120, Thailand; ^2^National Science and Technology Development Agency, 111 Thailand Science Park, Paholyothin Road, Khlong Nueng, Khlong Luang, Pathum Thani 12120, Thailand

## Abstract

The quality of images obtained from cone-beam computed tomography (CBCT) is important in diagnosis and treatment planning for dental and maxillofacial applications. However, X-ray scattering inside a human head is one of the main factors that cause a drop in image quality, especially in the CBCT system with a wide-angle cone-beam X-ray source and a large area detector. In this study, the X-ray scattering distribution within a standard head phantom was estimated using the Monte Carlo method based on Geant4. Due to small variation of low-frequency scattering signals, the scattering signals from the head phantom can be represented as the simple predetermined scattering signals from a patient's head and subtracted the projection data for scatter reduction. The results showed higher contrast and less cupping artifacts on the reconstructed images of the head phantom and real patients. Furthermore, the same simulated scattering signals can also be applied to process with higher-resolution projection data.

## 1. Introduction

X-ray cone-beam computed tomography (CBCT) has been widely used in many applications [1], especially for dental and maxillofacial applications [1, 2]. In typical CBCT, a gantry that consists of a cone-beam X-ray source and an area detector at the opposite side rotates 180–360 degrees around a patient's head to collect projection data. The main advantage of using a wide-angle cone-beam X-ray source and a large area detector includes a large field of view, thus reducing the scan time and the patient's radiation dose [2]. Nevertheless, the large area detector causes a larger amount of X-ray scattering signals than the smaller area detector [3]. These scattering signals reduce image quality of reconstructed images as appearing in terms of cupping artifacts, reduction of contrast, and streak artifacts [2–4].

There are several methods to reduce the X-ray scattering effect on the reconstructed images [4–15], such as using a beam stop array or a beam hole array to investigate the scattering signal pattern, point-spread function that describes scattering of the pencil beam X-ray to apply a deconvolution technique for the scatter artifact correction, and the Monte Carlo method to estimate the scattered X-ray distribution. From the fact that the X-ray interaction can be described as probability, the Monte Carlo method has become one of the popular methods to study X-ray scattering inside the object [10]. A lot of research used Monte Carlo simulation to estimate the intensity of the scattered X-rays, to consider the factors that affect amount of the scattered X-ray intensities, and to reduce the X-ray scattering effect on the reconstructed images [11–15]. The common procedure for scatter reduction was to subtract the scattering signals from the projection images [14, 15]. The results of this procedure showed better quality of the reconstructed images. In our recent experiment [16], Monte Carlo simulation based on Geant4 was used to reduce the effect of X-ray scattering inside a simple geometry object. The preliminary result showed that the procedure can improve the contrast and reduce the cupping artifact of the reconstructed images. Moreover, the CT numbers of the materials after the reduction were closer to the calculated values from the database.

In this paper, we modified our scatter reduction procedure to complex geometry objects, that is, real human heads. The dental CT system was simulated by using Geant4 and the head phantom as a reference object obtained from the DICOM images. The X-ray scattering signals from the simulation were used in the process to improve the experimental signals that were acquired by CT scanning of the head phantom and real human heads in various case studies. Because a patient head's size and a patient position of each case might differ from the simulated head phantom, a weighting factor was added in the scatter reduction process. The factor allows the simulated scattering signals of the head phantom to be used in many patient case studies without the appearance of new artifacts. The enhancement of reconstructed images after the scatter artifact reduction process is then analyzed.

## 2. Materials and Methods

### 2.1. The Dental CBCT System

**Figure 1:**
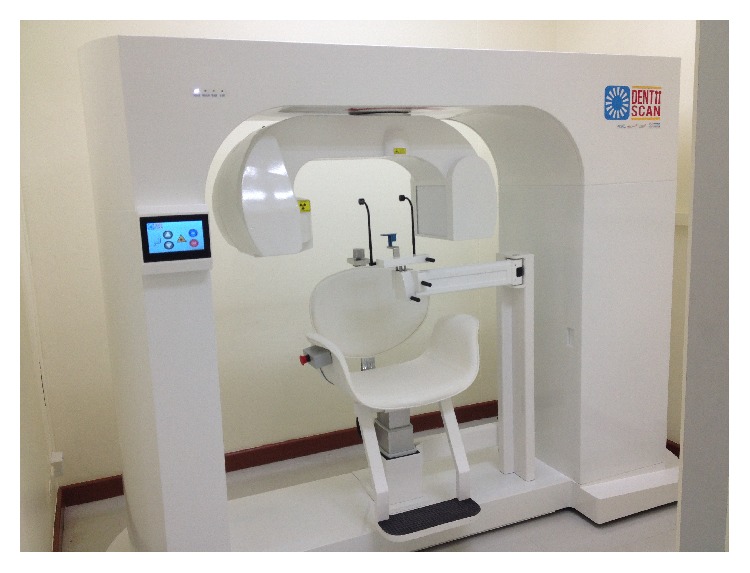
The dental CBCT scanner named DentiiScan 1.1.

**Figure 2:**
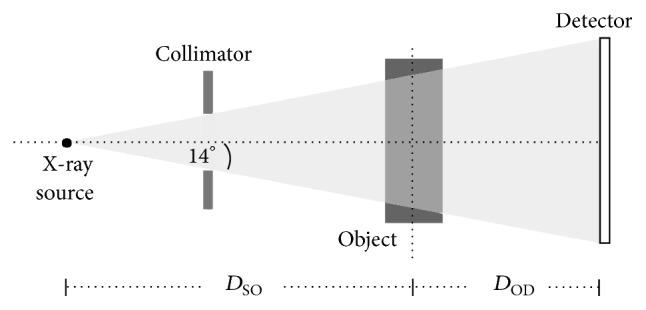
The CT system used in this study.

This study used the dental CBCT scanner named DentiiScan 1.1, which was developed in Thailand by the National Science and Technology Development Agency (NSTDA) (Figures 1 and 2) [17, 18]. The system consists of the cone-shaped X-ray source and the amorphous silicon flat panel detector (a-Si FPD) that rotates around a patient. The X-ray beam was generated by applying 90 kV voltage to an X-ray tube with a 14° tungsten anode and a 0.6 mm focal spot size. To reduce the patient dose, the low-energy X-rays that are not contributed to the receptor are suppressed by 10.6 mm Al equivalent filtration. The X-rays that pass through a patient are detected by the amorphous silicon flat panel detector with the CsI(Tl) scintillator (PaxScan 2520D Receptor, Varex Imaging Corporation, USA) with the size of 250 × 200 mm^2^ and the pixel pitch of 0.127 mm. The distance from the source to the object (*D*_SO_) was set at 482 mm and the distance from the object to the detector (*D*_OD_) was set at 226 mm. The image acquisition protocol we used in this study was 90 kVp and 108 mAs. 360 projection images were acquired from full rotation scan with 1° step angle. To collect those images, the positions of both head phantom and real patient heads were at the object location. The mid-transaxial plane was located at the occlusal plane, and the mid-sagittal plane was located along the middle of the phantom or the patient's face. The advantage of the large area flat panel used in the system allowed the large volumetric size of the reconstructed images with the maximum field of view (FOV) of about 160 × 130 mm^2^.

### 2.2. Geant4 Simulation

Geant4 is an open-source toolkit composed of C++ libraries for particle transport simulation which covers a wide range of energies. Thus, it is applicable to many fields, such as high energy, nuclear and accelerator physics, space science, and medical physics [19]. In this study, Geant4 version 9.4 was used to simulate the dental CBCT system to estimate the X-ray scattering signals that would later be used in the procedure of scatter artifact reduction. The electromagnetic processes that are defined in the simulation are Bremsstrahlung, ionization, and multiple scattering for electrons, as well as photoelectric effect, Compton scattering, and Rayleigh scattering for X-ray photons. These processes are based on the Penelope low-energy electromagnetic models.

**Figure 3:**
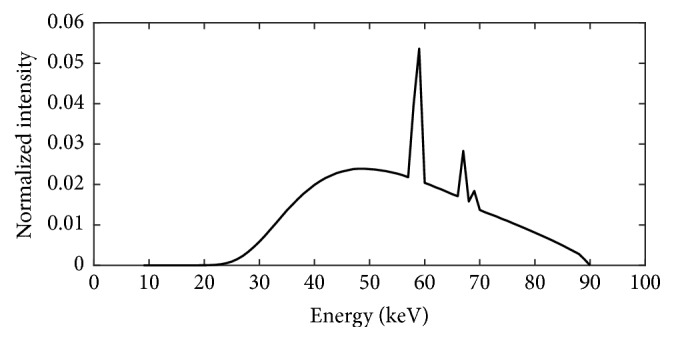
The energy spectrum of X-ray calculated using SpekCalc.

For primary particle generation in Geant4 simulation, the cone-beam X-rays can be created directly by defining their energy, position, and angular distributions. In this study, the energy distribution used as an input in Geant4 was the energy spectrum shown in Figure 3. This energy spectrum was calculated by SpekCalc version 1.1 [20], a program for calculating the X-ray emission spectra from tungsten anode X-ray tubes, using the same input parameters as the actual CBCT system, such as tube voltage, target angle, and X-ray filtration. Although the exact simulation of the X-ray tube structure was not directly applied, the energy spectrum from SpekCalc was further adjusted according to the half-value layer (HVL) of the actual system measured from the AGMS-D+ solid-state multisensor (Radcal Corporation, USA). The position distribution that was the starting positions of X-ray photons emission was defined as a circle surface with the diameter size of 0.6 mm. The directions of these X-ray photons were controlled by the angular distribution which was set to the isotropic type and limited by the maximum angle of 14° according to the tube anode.

**Figure 4:**
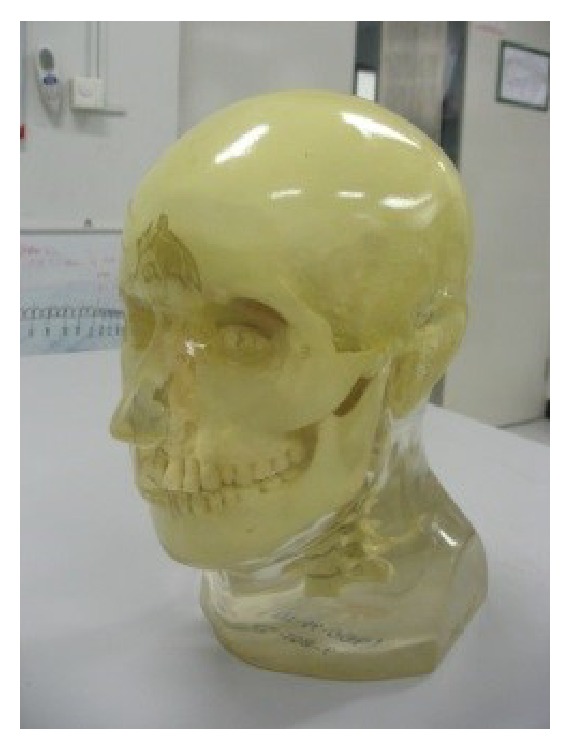
The anthropomorphic head phantom.

To imitate a patient's head, the object in this simulation was the anthropomorphic head phantom (model RS-108T, Radiology Support Devices, USA) as shown in Figure 4 which was constructed by reading the DICOM files of the reconstructed images [21]. The CT number in each pixel of the DICOM images was converted to the material density by using a CT number-density calibration curve. The obtained density value was assigned to its corresponding material type in that pixel; that is, the densities of air, soft tissue, and bone were defined in the range of 0–0.3 g/cm^3^, 0.3–1.2 g/cm^3^, and 1.2–1.95 g/cm^3^, respectively. These material properties were based on Geant4 material database. After all pixels were converted, the simulated object would correspond to the actual shape and density of the head phantom.

The simulated detector was modeled as a series of vacuum pixels with the pixel pitch of 0.508 mm and the total area size of 480 × 384 pixels. To acquire the simulation images, the number of X-ray photons that reached each pixel of the detector was directly counted. This detector performed as the ideal detector because the efficiency of photon conversion to the image data and electronic noise were neglected in the simulation. However, from our previous work [16], we compared the projection image from the simulation with the other from the actual CBCT scanner and the result showed that the profile of the simulated projection image agreed well with the experimental projection image.

To estimate the X-ray scattering signals from the objects, one important feature of Geant4 is its ability to track the particle. We can get the information of each particle step when it passes through the objects, such as its interaction, position, and energy. In this simulation, the X-ray photons that passed through the head phantom were tracked, and the number of interactions of each photon was counted. When the photons hit the detector, they were divided into two categories according to the results from X-ray tracking. The first category was the primary X-rays that did not interact with the object, while the other was the scattered X-rays that scattered inside the object. The output consisted of two projection image files that were the data of primary signals and the data of the scattering signals.

In this study, the simulation was run on Intel Core i7, 3.4 GHz, and 8 GB of RAM. Each projection image was simulated with 1.2 × 10^8^ primary photon particles, and the computation required about 110 minutes per projection. The output images were saved in the raw file format with the size of about 360 KB for one projection image.

### 2.3. Scatter Reduction Methods

**Figure 5:**
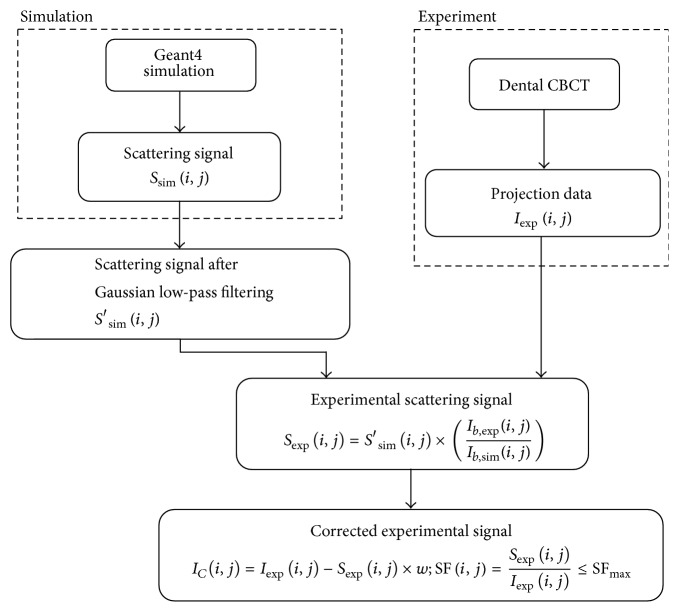
The process flow of the X-ray scatter reduction.

In simulation, the head phantom was used to represent any patient's head, and then the intensity of the simulated primary signal, *P*_sim_(*i*, *j*), and the intensity of the scattering signal after passing through the object, *S*_sim_(*i*, *j*), were estimated. From the advantage that scattered X-ray distribution has dominant low-frequency components, a slight difference of an object shape should not affect the scattering signals much; therefore, the simulated scattering signals are more suitable to use in the scatter reduction process instead of using the simulated primary signals. The overall procedure follows the process flow as shown in Figure 5. Due to high noise, the intensities of the simulated scattering signal were smoothened by applying a Gaussian low-pass filter. If the ratio of the scattered X-ray intensity to the blank-scan X-ray intensity from the simulation was assumed to be equal to that from the experiment, the intensity of the experimental scattering signal, *S*_exp_(*i*, *j*), can be obtained by the following equation:

(1) $$\begin{array}{c} S_{exp}\left(\;i,j\;\right)={S^{\prime }}_{sim}\left(\;i,j\;\right)\times \left(\;\frac{I_{b,exp}\left(i,j\right)}{I_{b,sim}\left(i,j\right)}\;\right), \end{array}$$

where (*i*, *j*) is the pixel coordinate at the detector, *S*′_sim_(*i*, *j*) is the intensity of the simulated scattering signal after applying a Gaussian low-pass filter, and *I*_*b*,exp_(*i*, *j*) and *I*_*b*,sim_(*i*, *j*) are the intensities of the X-ray signals without an object from the experiment and the simulation, respectively. According to the straightforward X-ray scatter reduction process, the corrected X-ray intensity, *I*_*C*_(*i*, *j*), corresponding to the experimental primary signal was typically derived from the measured intensity, *I*_exp_(*i*, *j*), subtracted by the intensity of the experimental scattering signal, *S*_exp_(*i*, *j*).

(2) $$\begin{array}{c} I_{C}\left(\;i,j\;\right)=I_{exp}\left(\;i,j\;\right)-S_{exp}\left(\;i,j\;\right). \end{array}$$

Since the intensity of the simulated scattering signal was acquired from the simulation of the CBCT system by using only the standard head phantom as a CT object, the intensity of the experimental scattering signal, estimated from (1), inside a large patient's head was assumed to be equal to that inside a small patient's head in a similar X-ray setting. Nevertheless, a large patient's head has X-ray attenuation more than a small patient's head; that is, the detected X-ray signals from a large patient's head are less than those from a small head. Directly using (2) to correct all of patient cases may cause the experimental data to be over- and undercorrected. This would cause additional artifacts on the reconstructed images. To overcome this problem, the intensity of the experimental scattering signal was modified by adding a weighting factor before scatter reduction. This factor enabled the simulated intensity of the X-ray that was scattered inside the head phantom to be used in the correction process for various human heads. Thus we can rewrite (2) as follows:

(3) $$\begin{array}{c} I_{C}\left(\;i,j\;\right)=I_{exp}\left(\;i,j\;\right)-S_{exp}\left(\;i,j\;\right)\times w, \end{array}$$

where *w* is the weighting factor, which is used to modify the intensities of the estimated scattering signal. Furthermore, the scatter fraction, SF(*i*, *j*), which is the ratio of the intensity of the scattering signal to the intensity of the measured signal, was limited by the maximum scatter fraction value, SF_max_. If the resulting SF(*i*, *j*) in the scatter reduction process has a value more than SF_max_, then the intensity of the scattering signal will be set to make the ratio equal to SF_max_ as in (4). The limit of the scatter fraction is useful to prevent overcorrection in the projection images [8].

(4) $$\begin{array}{c} SF\left(\;i,j\;\right)=\frac{S_{exp}\left(i,j\right)}{I_{exp}\left(i,j\right)}\leq {SF}_{max}. \end{array}$$

## 3. Results and Discussions

As the head phantom was used to represent any real human's head, we demonstrated the performance of our proposed method to reduce the effect of X-ray scattering on the reconstructed images using both the head phantom itself and the real patient data. In this study, the weighting factor value used in (3) was fixed at 0.8, and the maximum scatter fraction value was 0.8. These values were chosen by trial and error to reduce the scatter artifact in various cases with difference in both size and position of the patient's head, such that no additional artifacts appeared in the reconstructed images. In the first part of results, the projection data of the head phantom which is the same as the object in the simulation was used to verify the proposed reduction process. In the second part, the reduction process was applied to four real patient cases. The voxel size of the reconstructed images used in these parts was 0.4 mm, and the pixel size of the projection images was 0.508 mm (with total image size of 480 × 384 pixels) which is the same size as in the simulation. In the last part, the voxel size of the reconstructed images was changed to 0.2 mm, and the pixel size of the projection images was 0.254 mm (with total image size of 960 × 768 pixels), while the size of the simulated projection image was still 480 × 384 pixels. All reconstructed images used in this study were reconstructed by using our in-house software based on the FDK-based filtered back-projection method [22] with the Shepp-Logan filter and the cutoff frequency of 0.7 for the voxel size of 0.4 mm or 0.5 for the voxel size of 0.2 mm.

### 3.1. Scatter Reduction on the Head Phantom

**Figure 6:**
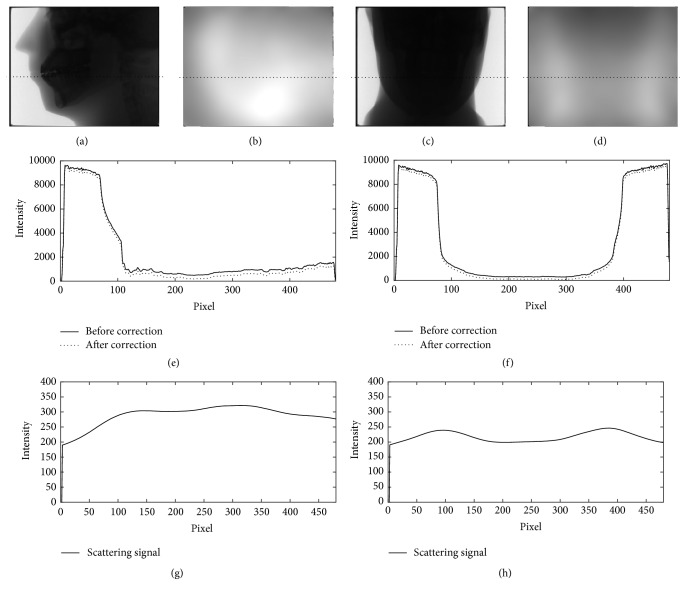
((a) and (c)) The projection images from the experiment, ((b) and (d)) the estimated scattered X-rays images, ((e) and (f)) the profiles of the projection images before (solid line) and after (dotted line) the scatter reduction process, and ((g) and (h)) the profiles of the estimated scattered X-rays.

**Figure 7:**
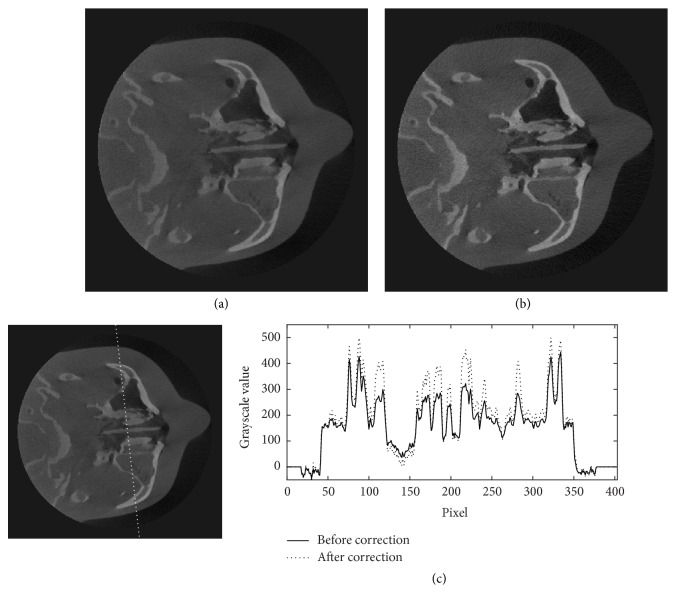
The reconstructed images of the head phantom at slice 39 of 300 (a) before and (b) after the scatter reduction process (window/level: 980/384); (c) the comparison of profile along the dotted line.

**Figure 8:**
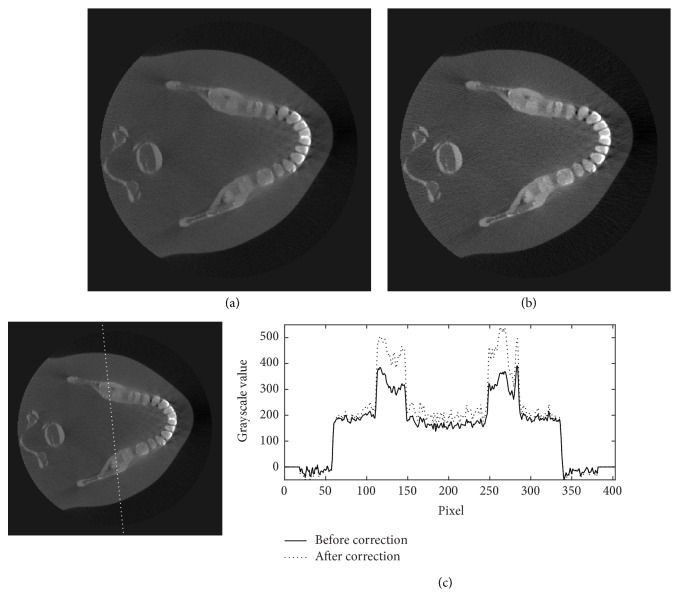
The reconstructed images of the head phantom at slice 149 of 300 (a) before and (b) after the scatter reduction process (window/level: 980/384); (c) the comparison of profile along the dotted line.

**Figure 9:**
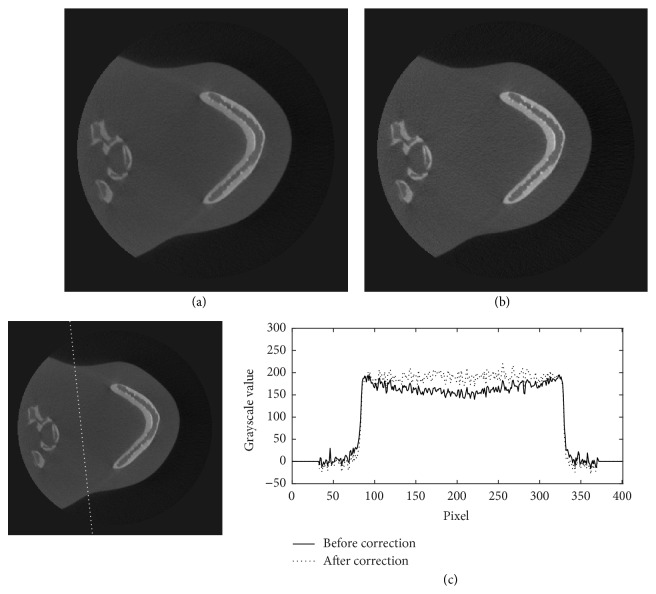
The reconstructed images of the head phantom at slice 195 of 300 (a) before and (b) after the scatter reduction process (window/level: 980/384); (c) the comparison of profile along the dotted line.

**Figure 10:**
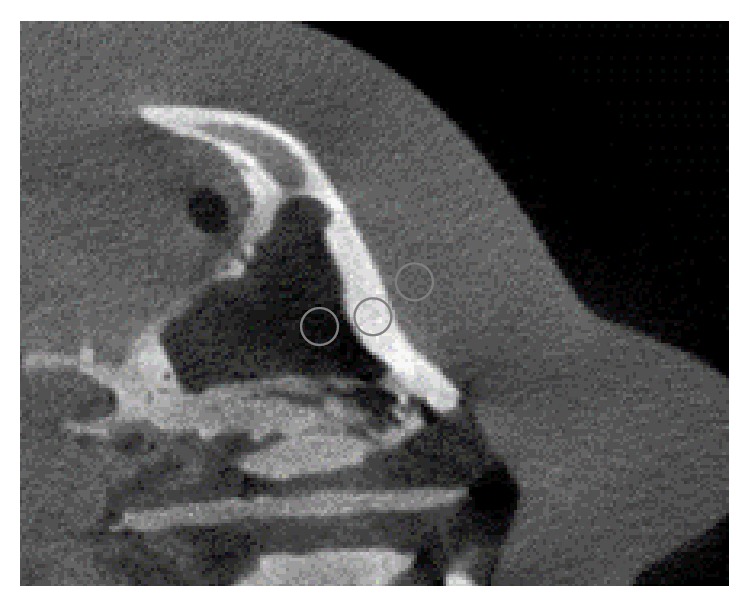
Selected regions for measuring grayscale values and contrast on the reconstructed image of the head phantom.

The projection data of the head phantom at two different view angles are shown in Figure 6. In this figure, the profiles along the dotted lines in Figures 6(a) and 6(c) are plotted in Figures 6(e) and 6(f), and their corresponding estimated scattered X-rays in Figures 6(b) and 6(d) are plotted in Figures 6(g) and 6(h), respectively. Figures 6(e) and 6(f) show that the X-ray intensities after the scatter reduction process appear to be lower than the ones before the process. Figures 7–9 show the reconstructed images before (Figures 7(a), 8(a), and 9(a)) and after (Figures 7(b), 8(b), and 9(b)) reduction of the X-ray scattering effect in three different axial slices having the same window/level. The comparisons of profiles before and after reduction process (Figures 7(c), 8(c), and 9(c)) show less cupping artifact and higher contrast in the corrected images. In Figure 9(c), the magnitude of cupping is reduced from 16.2% to only 0.1%. The reconstructed images with scatter reduction seem to have better contrast between soft tissues and bone. The comparison of contrast before and after scatter reduction in the selected regions in Figure 10 is shown in Table 1.

### 3.2. Scatter Reduction on Real Patients

In this part, the scatter reduction process was applied to four real patient cases. The scattering signal used for the scatter reduction process in this part also came from the simulation using the head phantom as an object like the previous part. The difficulty in this part arises from the fact that each patient has different size of his/her head and positioning might differ from the simulation. In each case, the angle of the head raw data was attempted to match between the actual and the simulation images as much as possible, while the factor about size variation of each head has already been included by the weighting factor in (3).

**Figure 11:**
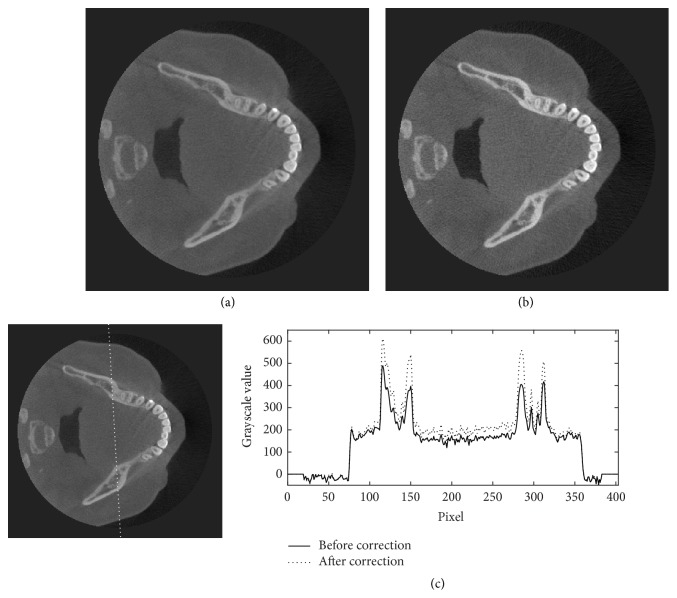
The reconstructed images of a patient's head with the size comparable with the head phantom (a) before and (b) after the scatter reduction process (window/level: 816/300); (c) the comparison of profile along the dotted line.

**Figure 12:**
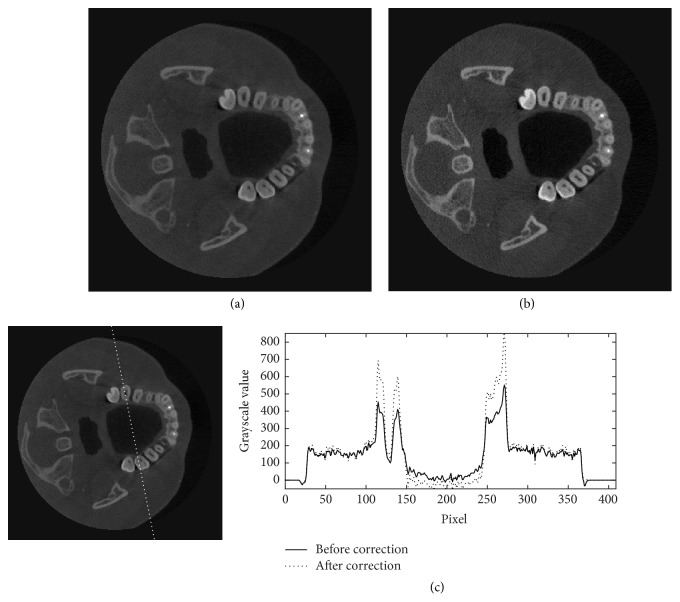
The reconstructed images of a large patient's head (a) before and (b) after the scatter reduction process (window/level: 1253/548); (c) the comparison of profile along the dotted line.

**Figure 13:**
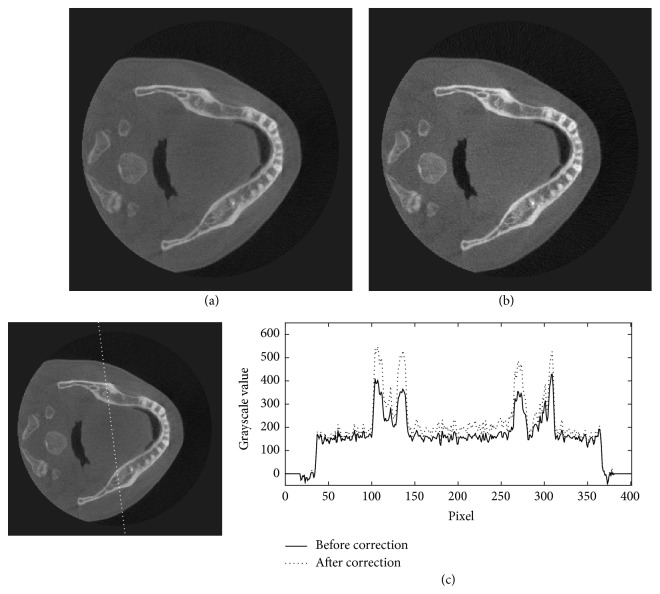
The reconstructed images of a small patient's head (a) before and (b) after the scatter reduction process (window/level: 796/308); (c) the comparison of profile along the dotted line.

**Figure 14:**
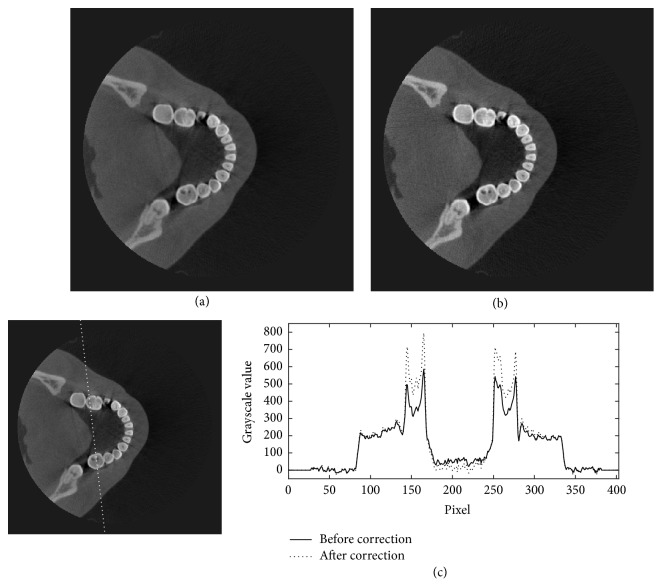
The reconstructed images of a patient's head with slightly backward positioning (a) before and (b) after the scatter reduction process (window/level: 1041/410); (c) the comparison of profile along the dotted line.

**Figure 15:**
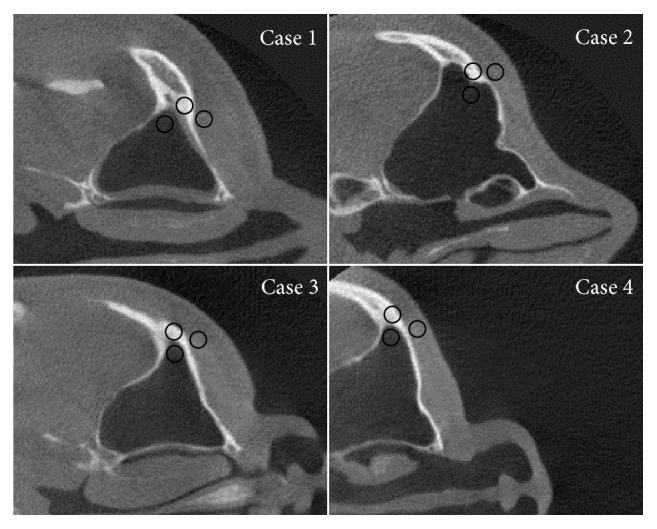
Selected regions for measuring grayscale values and contrast on the reconstructed image of real patients.

Figures 11–14 show the results of scatter reduction applied to four different real patient cases: (1) the head's size comparable with the head phantom, (2) the larger head, (3) the smaller head, and (4) the head with slightly backward positioning (about 4 cm). All of these images show that the contrast after reduction process is improved and the cupping artifact is decreased. The comparison of contrast before and after scatter reduction corresponding to the selected regions in Figure 15 is shown in Table 2. From the table, the absolute percentage change in case of the head size comparable with the head phantom appears to be a little higher than the other cases. However, for other cases, the contrast after scatter reduction is still increased about 16–25%.

### 3.3. Scatter Reduction on High-Resolution Reconstructed Images

**Figure 16:**
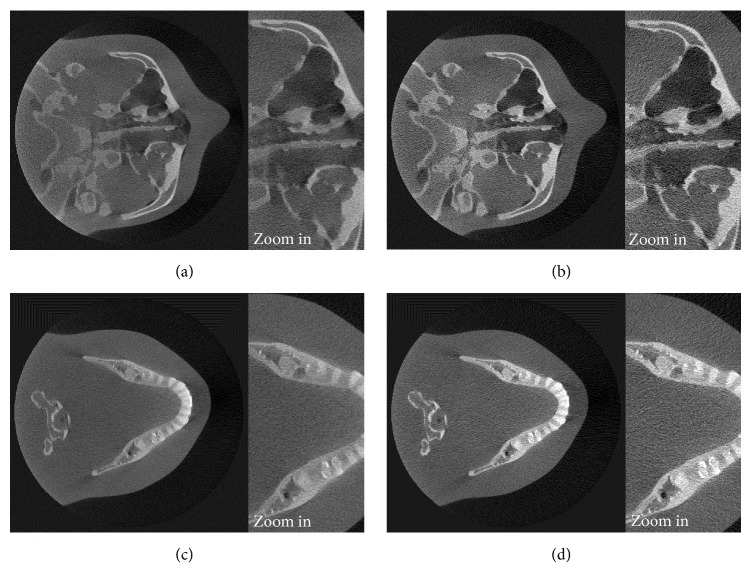
The reconstructed images of the head phantom in the 0.2 mm resolution mode before ((a) and (c)) and after ((b) and (d)) the scatter reduction process (window/level: 778/296).

**Figure 17:**
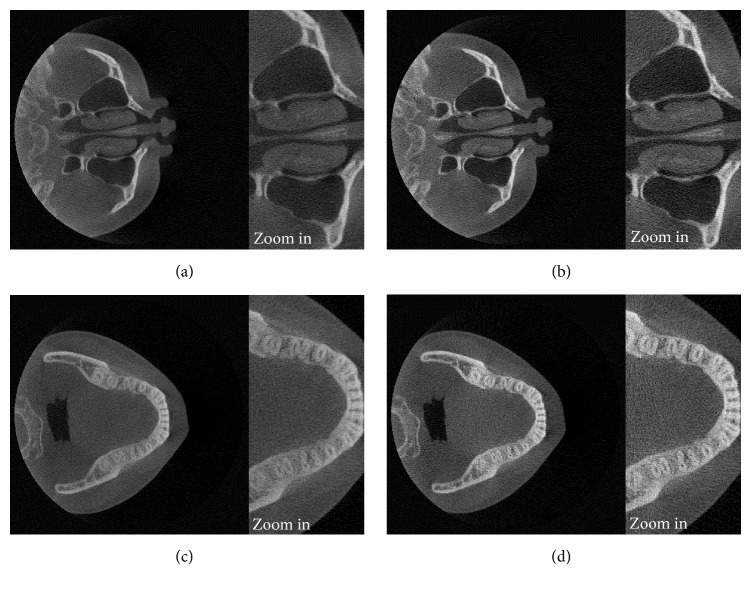
The reconstructed images of the patient's head in the 0.2 mm resolution mode before ((a) and (c)) and after ((b) and (d)) the scatter reduction process (window/level: 756/358).

**Figure 18:**
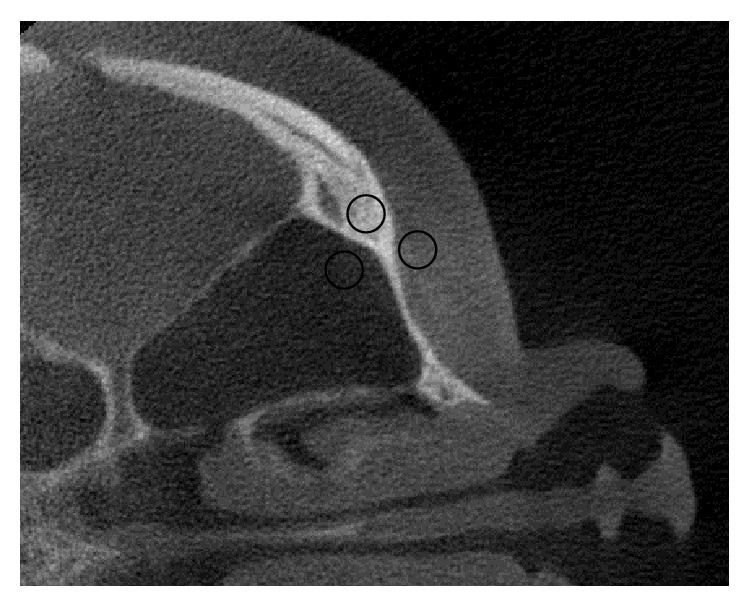
Selected regions for measuring grayscale values and contrast on the reconstructed image of the real patient with the voxel size of 0.2 mm.

In this part, we applied the simulated scattering signal to a larger image size of the projection data. The volume images with a voxel size of 0.2 mm were reconstructed from the projection images with the size of 960 × 768 pixels, while the same scattered X-ray data with the size of 480 × 384 pixels from the simulation as the previous part were still used in the scatter reduction process. This simulated scattering data were interpolated using the bicubic interpolation method before applying the scatter reduction process. Figures 16 and 17 show the high-resolution reconstructed images of the head phantom and the real patient case before and after scatter reduction, which improve the image quality. The comparison of contrast before and after scatter reduction corresponding to the selected regions as in Figures 10 and 18 is shown in Tables 3 and 4, respectively. The results in Table 3 were measured on the reconstructed image at the same slice as used in Table 1. The values show that the improvement of contrast of the images with the voxel of 0.2 mm is comparable with that of 0.4 mm in the previous part. In case of the real patient as shown in Table 4, the absolute percentage change has value in the same range as the value in case of voxel size of 0.4 mm. This verifies that the simulated scattering signals from the low-resolution projection image can also be applied to correct higher resolution data.

In this work, the weighting factor in the scatter reduction process was fixed to a suitable constant that did not cause additional artifacts in various patient cases. Nevertheless, each patient head has different sizes and positions, which may cause deviation in the experimental scatter estimates. To improve accuracy of the scatter estimation, the weighting factor should be varied, and the optimized weighting factor would be a challenging topic in the future. Although the flat filter was used in the simulation while the actual system used the bowtie filter, all results show reduction of X-ray scattering artifacts. However, to further improve scatter reduction, the bowtie filter should be taken into account in the simulation and studied in more detail. Furthermore, applicability of the proposed method to a small FOV is possible with adjustment of some parameters and the location of FOV. Since the narrow-collimated X-ray is often applied, the effect of various collimator sizes on the scattering signals should be further studied.

## 4. Conclusions

In this paper, we proposed the Monte Carlo method based on Geant4 to reduce the scatter artifact in CBCT images by modeling the actual dental CBCT system. For simulation, the scattering signals of the transmitted X-rays through the head phantom were estimated. These simulated scattering signals were modified and subtracted from the experimental intensity data to reduce the effect of X-ray scattering on the reconstructed images. We evaluated our proposed scatter reduction method on the head phantom itself as well as the real patient data. Although the alignment of head phantom in the actual CBCT system might not match perfectly with the simulation system, this does not affect scatter reduction much because the scattered X-ray distribution has dominant low-frequency components. The reconstructed images after the reduction process show large improvement of contrast and cupping artifact reduction. Similarly, the real patient data with scatter reduction show better image quality in the reconstructed images as well. Lastly, the same scattered X-ray data from the simulation with the low-resolution projection images were interpolated and then applied to a larger image size of the projection data to reduce the X-ray scattering effect on the high-resolution reconstructed images. The reconstructed images of the head phantom and the patient's head show that this process is applicable for scatter reduction on higher-resolution projection data as well.

## Figures and Tables

**Table 1:** Comparison of contrast in the selected regions and the percentage of cupping before and after scatter reduction on the reconstructed images of the head phantom.

Reconstructed images	Contrast (grayscale value)		Cupping in soft tissue (%)
	Air bone	Bone tissue	
Before scatter reduction	358.13	221.65	16.20
After scatter reduction	479.35	304.13	0.10
Absolute percentage change	33.85%	37.21%	99.38%

**Table 2:** Comparison of contrast in the selected regions before and after scatter reduction on the reconstructed images of real patients.

Patient case	Reconstructed images	Contrast (grayscale value)	
		Air bone	Bone tissue
Case 1	Before scatter reduction	384.35	261.97
	After scatter reduction	473.62	333.42
	Absolute percentage change	23.22%	27.27%

Case 2	Before scatter reduction	405.72	262.71
	After scatter reduction	471.49	309.16
	Absolute percentage change	16.21%	17.68%

Case 3	Before scatter reduction	378.94	223.56
	After scatter reduction	465.01	280.51
	Absolute percentage change	22.71%	25.47%

Case 4	Before scatter reduction	393.35	238.34
	After scatter reduction	458.49	287.16
	Absolute percentage change	16.56%	20.48%

**Table 3:** Comparison of contrast in the selected regions and the percentage of cupping before and after scatter reduction on the reconstructed image of the head phantom with the voxel size of 0.2 mm.

Reconstructed images	Contrast (grayscale value)		Cupping in soft tissue (%)
	Air bone	Bone tissue	
Before scatter reduction	359.00	221.77	20.70
After scatter reduction	484.28	309.09	0.61
Absolute percentage change	34.90%	39.37%	97.05%

**Table 4:** Comparison of contrast in the selected regions before and after scatter reduction on the reconstructed images of the real patient with the voxel size of 0.2 mm.

Reconstructed images	Contrast (grayscale value)	
	Air bone	Bone tissue
Before scatter reduction	356.91	262.01
After scatter reduction	445.87	339.56
Absolute percentage change	24.93%	29.60%
